# The microbial biosphere of the coral *Acropora cervicornis* in Northeastern Puerto Rico

**DOI:** 10.7717/peerj.3717

**Published:** 2017-08-29

**Authors:** Filipa Godoy-Vitorino, Claudia P. Ruiz-Diaz, Abigail Rivera-Seda, Juan S. Ramírez-Lugo, Carlos Toledo-Hernández

**Affiliations:** 1 Department of Natural Sciences, Microbial Ecology and Genomics Lab, Inter American University of Puerto Rico, San Juan, PR, USA; 2 Department of Environmental Sciences, University of Puerto Rico Rio Piedras Campus, San Juan, PR, USA; 3 Sociedad Ambiente Marino, San Juan, PR, USA; 4 Department of Biology, University of Puerto Rico Rio Piedras Campus, San Juan, PR, USA

**Keywords:** Coral, 16S rDNA, Caribbean, Microbiota, Depth-related

## Abstract

**Background:**

Coral reefs are the most biodiverse ecosystems in the marine realm, and they not only contribute a plethora of ecosystem services to other marine organisms, but they also are beneficial to humankind via, for instance, their role as nurseries for commercially important fish species. Corals are considered holobionts (host + symbionts) since they are composed not only of coral polyps, but also algae, other microbial eukaryotes and prokaryotes. In recent years, Caribbean reef corals, including the once-common scleractinian coral *Acropora cervicornis*, have suffered unprecedented mortality due to climate change-related stressors. Unfortunately, our basic knowledge of the molecular ecophysiology of reef corals, particularly with respect to their complex bacterial microbiota, is currently too poor to project how climate change will affect this species. For instance, we do not know how light influences microbial communities of *A. cervicornis*, arguably the most endangered of all Caribbean coral species. To this end, we characterized the microbiota of *A. cervicornis* inhabiting water depths with different light regimes.

**Methods:**

Six *A. cervicornis* fragments from different individuals were collected at two different depths (three at 1.5 m and three at 11 m) from a reef 3.2 km off the northeastern coast of Puerto Rico. We characterized the microbial communities by sequencing the 16S rRNA gene region V4 with the Illumina platform.

**Results:**

A total of 173,137 good-quality sequences were binned into 803 OTUs with a 97% similarity. We uncovered eight bacterial phyla at both depths with a dominance of 725 Rickettsiales OTUs (Proteobacteria). A fewer number (38) of low dominance OTUs varied by depth and taxa enriched in shallow water corals included Proteobacteria (e.g. *Rhodobacteraceae* and* Serratia*) and Firmicutes (*Streptococcus*). Those enriched in deeper water corals featured different Proteobacterial taxa (Campylobacterales and Bradyrhizobium) and Firmicutes (*Lactobacillus*).

**Discussion:**

Our results confirm that the microbiota of *A. cervicornis* inhabiting the northeastern region of Puerto Rico is dominated by a Rickettsiales-like bacterium and that there are significant changes in less dominant taxa at different water depths. These changes in less dominant taxa may potentially impact the coral’s physiology, particularly with respect to its ability to respond to future increases in temperature and CO2.

## Introduction

Coral reefs cover only 0.1% of the ocean’s floor, yet they host one quarter of the total biodiversity of the oceans. The variable shapes and heavily calcified skeletons that characterize the corals themselves create a three-dimensional seascape that provides myriad niches for organisms belonging to virtually all phyla of the animal kingdom, as well as other eukaryotic kingdoms (Holbrook et al., 2015). Scleractinian corals also harbor great prokaryotic diversity distributed across the coral’s many micro-niches, e.g., within the mucus, soft tissue and skeleton (Ainsworth et al., 2015). The roles of coral-associated microbes appear to be critical for coral homeostasis, health and protection against disease, to the extent that the coral host and the associated microbial communities are generally considered as a single functional unit, termed the coral “holobiont” (Thompson et al., 2014). Culture-dependent analyses of coral mucus revealed that bacterial diversity is between 100 and 1,000 fold greater than that of the surrounding seawater (Rosenberg et al., 2007).

Furthermore, coral-associated microbes tend to be species-specific, meaning that individuals from the same coral species have similar bacterial and archaeal communities, even when these individuals are several kilometers apart. In contrast, different coral species living in close proximity have different bacterial communities (Morrow et al., 2012). Compelling evidence suggests that most of the coral-associated bacteria are undescribed, as many 16S rRNA sequences from scleractinian corals have a low match or no match at all with 16S sequences available in the NCBI database (Rohwer et al., 2002; Frias-Lopez et al., 2008; Sun, Anbuchezhian & Li, 2016). Additionally, coral-associated bacteria are rare in the sense that many phylotypes are present in very low abundance (species relative abundance <0.1%) in the coral holobiont which are likely to be functionally relevant as in many other ecosystems (Lynch & Neufeld, 2015; Hausmann et al., 2016; Jousset et al., 2017). In fact, the phenomena of a rare microbiota associated with corals has been observed in different coral species across the world (Sunagawa et al., 2009; Lee et al., 2012; Bayer et al., 2013; Ainsworth et al., 2015).

It is generally accepted that global climate change is having a great impact on ocean waters. Shallow seawater has warmed by approximately 0.4 °C during the past five decades while solar radiation is also on the rise, partly because of ozone layer depletion (Levitus et al., 2009). Coral bleaching and disease have both been associated with increased water temperature and solar radiation; therefore, areas within reefs of reduced water temperatures or light irradiance relative to the surroundings might be more supportive of coral health. In fact, bleaching and bleaching-related mortality has been reported to be significantly higher in coral from shallow waters than in corals at deeper waters (Bridge et al., 2014).

Coral-prokaryote symbioses are seemingly complex and sustained under a narrow range of tolerance. For instance, a metagenomics analysis of the finger coral *Porites compressa* revealed that colonies exposed to multiple stressors, including acute thermal stress, displayed rapid and significant shifts in the taxonomic structure of the coral-associated microbiota with significant functional and physiological consequences (Vega Thurber et al., 2009). Although revealing, this study was conducted by placing collected corals in an aquarium under conditions that are unlikely to be observed naturally. Therefore, exactly how environmental changes in temperature and solar radiation could impact coral-prokaryote symbiosis is yet to be concisely determined. Nevertheless, because of the large role played by light and temperature on coral health, we hypothesize that the microbial community structure of *Acropora cervicornis* at reef depths with different light and temperature regimes may be considerably different. Deep sequencing of 16S rRNA genes with next-generation platforms has revealed a high bacterial diversity in invertebrate hosts and, in the case of corals, the presence of bacterial genera frequently detected across their geographic distribution (Ainsworth et al., 2015). The ever-increasing resolution provided by recent sequencing technologies has revealed a diverse collection of microbes of low relative abundance, which is termed the “rare biosphere” (Sogin et al., 2006).

With this study, we aimed to understand depth-related differences in microbial assemblages in the scleractinian coral *A. cervicornis* in northeastern Puerto Rico. This species was selected as the study model because it is emblematic of what is occurring in Caribbean coral reef systems. This coral was once among the predominant reef-building corals in the Caribbean, but over 97% of populations across the Caribbean have collapsed due to environmental stressors such as temperature-induced bleaching and diseases (Aronson & Precht, 2001). The dire situation faced by *A. cervicornis* across the Caribbean region has led the US National Marine Fishery Service and the Union for Conservation of Nature to list *A. cervicornis* on the US Endangered Species Act (ESA) and Red List of Critically Threatened Species respectively. Hence, understanding how the natural environmental variation due to differences in depth may affect the microbial assemblages of *A. cervicornis* will shed light on how this species will cope with future changes in seawater quality brought on by climate change. To gain insight into how environmental differences affect coral microbial communities, we sampled *A. cervicornis* specimens at two depths and profiled their bacterial communities.

## Materials and Methods

### Coral samples

**Figure 1:**
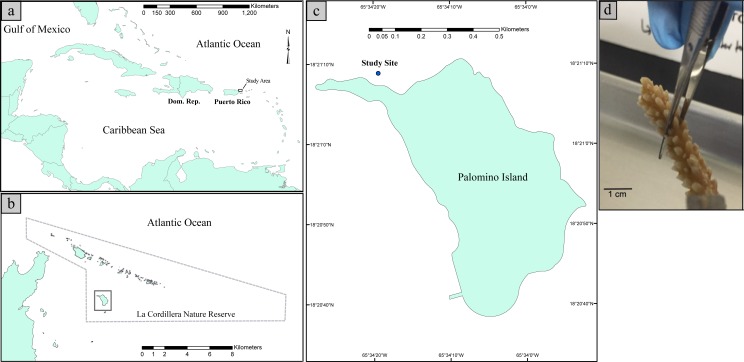
Map illustrating the geographic position of Puerto Rico in the Caribbean Basin (A). Map illustrating the study site with respect to La Cordillera Natural Reserve (B). Map illustrating the sampling site with respect to Palomino Island (18°21′10.8″N 65°34′24.4″W) (C). Picture of a collected fragment of *Acropora cervicornis* prior to DNA extraction (D).

Coral sampling was conducted within the La Cordillera Natural Reserve (LCNR), specifically at the northern shores of Isla Palomino, which is located 3.2 km from the northeastern coast of Puerto Rico (18°21′10.8″N 65°34′24.4″W, Fig. 1). The sampling site is a low topographic relief, fringing reef with relatively clear waters year-around and moderate to high wave energy depending on the water depth. Due to the reef’s northeastern orientation, the site is exposed to easterly trade winds. In addition, given the absence of perennial freshwater streams in Palomino Island, concomitant with the distance from the Fajardo watershed, (the nearest freshwater system is ∼6 km west of the site), coastal waters are mostly free of terrestrial sediments and thus have a horizontal water visibility well over 10 m. The corals assemblage at the collection site is dominated by gorgonian corals such as *Gorgonia ventalina* and *Pseudopterogorgia aerosa* and small colonies of the scleractinian corals *Orbicella annularis*, *Acropora palmata*, *Porites astreoides* and spread *Acropora cervicornis* clusters composed of several individuals. For a further description of the study area please see Hernandez-Delgado et al. (2006), Mercado-Molina et al. (2015) and Ruiz-Diaz et al. (2016).

Within this site, six *A. cervicornis* colonies were collected in August of 2015. Three of them were collected at a depth of 1.5 m (hereafter “shallow” samples) and three from 11 m depth (hereafter “deep” samples). Shallow samples were collected from the reef crest and were at least 5–6 m apart from each other. Corals of the reef crest are nearly continuously exposed to relatively high water motion, and reef crest temperature averages ∼29 °C. Solar radiation was ∼11,203.55 Lux (SI unit of illuminance = 1 lumen/m2) at the time of sampling. Deep samples were collected from at least 8–10 m apart within the back-reef zone. This zone is characterized by lower water motion and wave action, and average temperature is ∼28 °C. Solar radiation was ∼3,429.36 Lux at the time of collection. Temperature and light intensities were estimated by placing one Hobo Pendant temperature/light data logger 64k-UA-002-64 (Onset Company) per depth at the study site at the time of collection. Although we do not have multiple log readings per site, a previous study conducted by the current authors in the same collection sites found that the temperature and light were significantly different between the two depths (Ruiz-Diaz et al., 2016). Collected fragments were 6 cm in length from the tip of the colonies. Each fragment was individually placed in a sterile vial while underwater and the vials containing the coral fragments were put in dry ice once they reached the surface. Samples were stored at −80 °C until processing. Sampling was approved by the Department of Natural and Environmental Resources of Puerto Rico permit number DRNA: 2016-IC-175 issued to Carlos Toledo-Hernández.

### DNA extraction

Coral samples were prepared for DNA extraction by scraping the surfaces of the tips (top 1.5 cm) of the branches (see Fig. 1) with sterile scalpel blades. This resulted in ∼400 mg of mucus, tissue and some skeletal material for each samples. The scraped material was subsequently processed using the PowerSoil DNA isolation kit (MO BIO, Carlsbad, CA, USA) following the manufacturer’s specifications. To increase DNA yield, a homogenization step was performed by mixing the lysate with beads using a PowerLyzer™ 24 Bench Top Bead-Based Homogenizer (MO BIO, Carlsbad, CA, USA) for 2 min at 2,000 rpm. Additionally, a second DNA extraction was done using the resultant pellet formed after the first homogenization step of the DNA extraction. Both DNA extractions (from the tissue and the tissue fragments in the bead tube) were pooled. Genomic DNA quality control was assessed using agarose gel electrophoresis with DNA standards of known molecular weight and concentration yielding 20–30 ng gDNA per sample. No further DNA purification steps were performed as PowerSoil DNA isolation kits are known to be effective at removing PCR inhibitors (Santos et al., 2012).

### 16S rRNA gene PCR and amplicon deposition

The V4 hypervariable region of the 16S ribosomal RNA was amplified by PCR using the universal bacterial and archaeal primers: 515F (5′ GTGCCAGCMGCCGCGGTAA 3′) and 806R (5′ GGACTACHVGGGTWTCTAAT 3′) as used with the Earth Microbiome Project (Caporaso et al., 2012). Amplification conditions were 1 cycle of 94  °C for 3 min, 35 cycles of 94 °C for 45 s, 50 °C for 60 s, 72 °C for 90 s and a final extension of 72 °C for 10 min. The six amplicons of ∼300 bp were barcoded to allow for sample multiplexing and paired-end sequenced in the Illumina MiSeq platform at the Sequencing and Genotyping Facility of the University of Puerto Rico. The resulting demultiplexed raw sequences per sample, as well as the 803 16S rRNA gene sequence representatives of the operational taxonomic units (OTUs) per sample were deposited in the NCBI BioProject ID PRJNA379103 with SRA accession SRP102061.

### Community profiling and bioinformatics

Demultiplexed reads underwent quality control using QIIME (Kuczynski et al., 2012), selecting those reads with Phred scores > 20 (99% confidence) and lengths > 200 bp which were searched for chimeras with the usearch61 hierarchical clustering method (Edgar, 2010). Sequences were binned into OTUs in QIIME using *de novo* OTU assignment methods (thus the “*de novo*” ids for OTUs), with a 97% sequence similarity with Greengenes core representative sequences Gg_13_8_99.taxonomy (McDonald et al., 2011). The algorithm chooses an OTU “centroid sequence” to be the representative sequence for each OTU. Sequence alignment was done using the Python nearest alignment space termination tool (PyNAST), and taxonomy assignment was done with the uclust consensus taxonomy assigner using QIIME’s default settings (Kuczynski et al., 2012). Chloroplast and mitochondria OTUs were removed from downstream analyses using the script filter_taxa_from_otu_table available in QIIME (Kuczynski et al., 2012). Additionally, stringent OTU filtering included the removal of singletons and OTUs with less than two sequences per sample to eliminate overestimation caused by sequencing artifacts. Data analyses including diversity estimates and taxonomic composition of the coral samples was done using QIIME 1.9.1 (Kuczynski et al., 2012) after data was subsampled with a rarefaction level of ∼28,000 sequences per sample, to mitigate bias in the analyses due to differences in sampling depth. Alpha diversity was calculated using the phylogenetic diversity metric of Faith (PD) to assess community diversity of the samples. Abundance was not taken into account, but rather the branch lengths of the phylogenies connecting all species to each community (Faith, 1992). Significant differences in the rarefaction curves were calculated with a non-parametric two-sample *t*-test using 999 Monte Carlo permutations using the QIIME script compare_alpha_diversity.py. Beta and alpha diversity analyses, as well as core microbiome analyses, were done through QIIME (Kuczynski et al., 2012). Taxa summaries were built by modifying the QIIME L2 and L6 taxonomy tables with the R package reshape2 (Wickham, 2007).

To explain differences among microbial communities inhabiting shallow and deep corals, we used principal coordinates analysis (PCoA) on UniFrac distances, a beta-diversity measure that uses phylogenetic information to compare samples (Lozupone, Hamady & Knight, 2006). Statistical analyses on beta diversity were made using ANOSIM, a non-parametric statistical test that compares ranked beta diversity distances between different group depths found in the mapping file and calculates a *p*-value based on the unweighted Unifrac table used to generate the 3D PCoA plots (Kuczynski et al., 2012). The test was done using the script compare_categories.py in QIIME (Kuczynski et al., 2012) with the Unifrac distance matrix as the input file. Unweighted UniFrac PCoA biplots were visualized in the EMPeror Visualization Program (Vazquez-Baeza et al., 2013). Additionally, beta diversity analyses using non-metric multidimensional scaling (nMDS) ordinations of Bray Curtis dissimilarity was done using distance metrics computed from the rarefied OTU table and the metadata. We used nMDS ordination, achieved by the *metaMDS* wrapper function from the vegan package in R (Oksanen et al., 2008). The ordination was applied such that the data was scaled down to two dimensions.

The same analysis was done to understand the depth of diversity of the dominant Rickettsiales OTUs. A species table was prepared, and OTUs were filtered to retain only Rickettsiales OTUs; then, the resulting OTUs were compared between the two sampling depths. To analyze species composition similarities across sites a Principal Coordinate Analysis (PCoA) ordination was used. The PCoA was constructed using a UniFrac distance matrix and visualized through QIIME. Additionally, we used a log-likelihood ratio to test which OTUs changed significantly in relative abundance between the two depths. This test compares the ratios of the OTU frequencies in the sample groups to an “extrinsic hypothesis” that assumes that all sample groups have equal OTU frequencies, thus revealing which taxa have significantly different OTU frequencies.

An alpha rarefaction plot was built for richness estimations using the Chao 1 values. These values represent the estimated true species richness of a sample and are calculated through the workflow script for performing alpha rarefaction in QIIME that in turn implements the Chao 1 abundance-based estimator (Chao, 1987). An alpha rarefaction plot was then built for the Chao 1 richness estimations between microbial communities in deep and shallow coral samples. Additionally, we used a non-parametric two-sample *t*-test statistical with Monte Carlo permutations to compare the richness curves between shallow and deep group samples.

OTUs that changed significantly between the two sample categories were found using the script group_significance.py through the implementation of a G-test (log-likelihood ratio test) that compares the frequency of OTUs across all samples and finds those that are significantly different between both groups. Those significantly different OTUs (*p* < 0.05) were grouped into an OTU table that underwent DESeq2 negative binomial Wald normalization for visualization purposes as the numbers of individuals per sample greatly varied. This normalization step was implemented in QIIME using the script normalize_table.py. The normalization in QIIME uses a variance stabilization transformation function *(VST*) that is applied to the count data, as described in https://www.rdocumentation.org/packages/DESeq/versions/1.24.0/topics/varianceStabilizingTransformation. The heatmap showing the taxa that significantly differed in abundance between depths (*p* < 0.05) was built using the heatmap.3 function in R (Zhao et al., 2014). Significant taxa were further highlighted using boxplots made with the vegan package in R (Oksanen et al., 2008). A detailed repository and tutorial with all the necessary intermediary files and scripts to serve as a guide to generate the plots and perform the analyses used in this publication is available on GitHub: https://github.com/meglab2017/The-microbial-biosphere-of-the-coral-Acropora-cervicornis-in-Northeastern-Puerto-Rico.

## Results

### Alpha and beta diversity estimates of corals at different depths

Coral individuals collected at 1.5 m (shallow samples 1, 2 and 3 hereafter) received more solar radiation compared to those at 11 m (deep samples 1, 2 and 3 hereafter). The total number of raw reads was 715,825 for the six samples. A total of 173,137 sequences were used for analysis, in which the average ± standard deviation of the number of reads was 29,135 ± 177.4 for the shallow samples and 28,578 ± 368 for deep samples. These sequences correspond to a subsampling to a rarefaction level of 28,000 sequences, without a replacement, thus guaranteeing an unbiased analysis (Table 1). The binning of the 173,137 high quality reads resulted in 803 operational taxonomic units (OTUs) (Table 1, Table S1).

**Figure 2:**
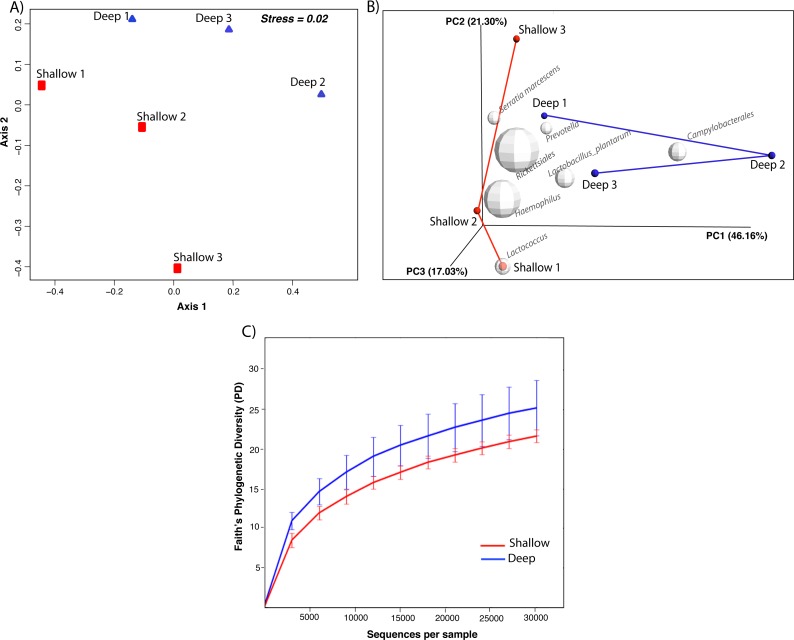
Non-metric multidimensional scaling (nMDS) ordinations of Bray Curtis dissimilarity between the bacterial communities inhabiting shallow and deep corals (A). Beta diversity 3D PCoA plot based on Unifrac distance matrix. Superimposed on the PCoA plot are gray spheres indicating the most abundant bacterial taxa associated with shallow and deep corals. The sizes of the spheres represent the relative abundance of the taxon and the location of the spheres within the plot indicate sample-specific associations (B). Alpha diversity curves for Faith’s PD index comparing shallow and deep corals. Error bars in the figure correspond to one standard deviation out from the average (*n* = 3 biological replicates/depth) (C).

nMDS ordination based on the relative dissimilarities of the samples (Bray Curtis) shows that shallow samples have higher dispersion and are separated from the deep samples by axis 1 (Fig. 2A). PCoA revealed that the first principal component, sample origin, represented the highest variance (PC1 = 46.16%, Fig. 2B) indicating that bacterial communities from coral samples partition according to sampling depth. Nonetheless, the non-parametric two-sample *t*-test ANOSIM revealed non-significant differences (Rstat = 0.59; *p* = 0.221) thus demonstrating that the similarity between groups is not significantly greater than the similarity within each of the sample groups. In addition, alpha diversity measures revealed no significant differences in phylogenetic diversity (*t*-stat = 0.548; *p* = 0.891, Fig. 2C). When visualizing the most abundant bacterial taxa associated with each sample, biplot indicated that Rickettsiales, *Serratia marcescens* and *Lactococcus* were most likely to be found in shallow coral samples, while *Prevotella*, *Lactobacillus* and Campylobacterales were more likely associated with the deep samples (Fig. 2B). Taken together, these results indicate that the species diversity did not vary significantly within each sampling depth and that there were only subtle differences in diversity between shallow and deep samples.

**Table 1:** The microbial biosphere of the coral *Acropora cervicornis* in Northeastern Puerto Rico. Number of sequences and OTU estimates across samples.

Sample ID	Depth (m)	Total number of raw sequences	Number of sequences used in the analyses	Number of OTUs (from a total of 803)
Shallow 1	1.5	171,429	29,076	435
Shallow 2	1.5	55,892	29,334	520
Shallow 3	1.5	29,886	28,994	492
Deep 1	11	283,069	28,308	542
Deep 2	11	77,290	28,428	413
Deep 3	11	98,259	28,997	386

### Taxonomic profiles of the *A. cervicornis* microbiome at different depths

**Figure 3:**
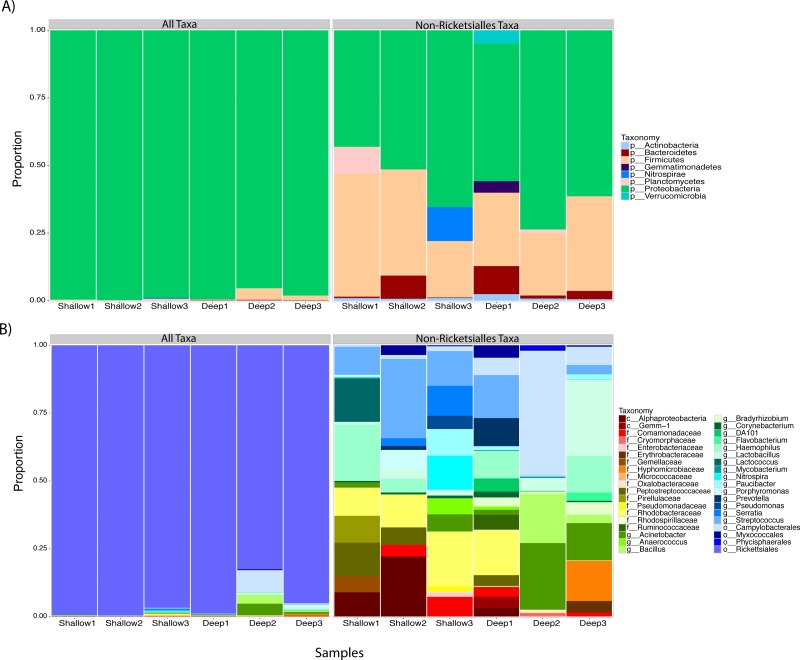
Taxonomic profiles at the phyla-level (A) and genus-level (B). Panels depict OTU tables including (“All Taxa”) and excluding Rickettsiales OTUs (“Non-Rickettsiales Taxa”).

We proceeded to explore the taxonomic profiles of the *A. cervicornis*-associated microbiome at different depths. At the phyla-level, our community profile analysis showed a total of eight phyla, with a dominance of Proteobacteria (specifically from the order Rickettsiales) at both sampling depths (95%; Fig. 3A, left panel). When these dominant Rickettsiales OTUs were filtered out of our analysis, other taxa from the Proteobacteria phylum dominated at both sampling depths (∼55%) followed by the phyla Firmicutes (∼35%), Bacteroidetes (∼5%) and Actinobacteria (∼2%) (Fig. 3A, right panel). Despite the dominant taxa being shared by corals from both sampling depths, some low dominance phyla appeared exclusively at only one of the sampling depths. Two phyla, Planctomycetes and Nitrospira, were only observed in the shallow samples, whereas Gemmatimonadetes and Verrucomicrobia were uniquely observed in deep samples (Fig. 3A, right panel).

The community profile analysis at the genus level revealed from the rarefied analyses revealed a total of 38 different genus-level OTUs, 19 of them were shared between shallow and deep samples, while 8 and 11 were exclusively isolated from shallow and deep samples respectively (Fig. 3B). Similar to the phylum-level analysis, most of these genus-level OTUs belonged to Proteobacteria and Rickettisales (95%). A total of 725 Rickettsiales-like OTUs were dominant across all samples independently of sampling depth. An analysis of the 78 non-Rickettsiales OTUs (<5%) revealed that the taxa most likely to differ in abundance between depths were low abundance Proteobacteria (Fig. 3B, left panel). In fact, nearly 60% of these were *Haemophilus, Acinetobacter,* Rhodobacteraceae, *Serratia, Pseudomonas*, Campylobacterales or other unclassified Proteobacteria (Fig. 3B, left panel). The other dominant phylum in the non-Rickettsiales group was Firmicutes (∼18%), with the most dominant genus being, *Lactococcus* and Peptostreptococcaceae mostly in the shallow samples while *Lactobacillus*, *Anaerococcus* and *Bacillus* were most dominant in the deep samples.

**Figure 4:**
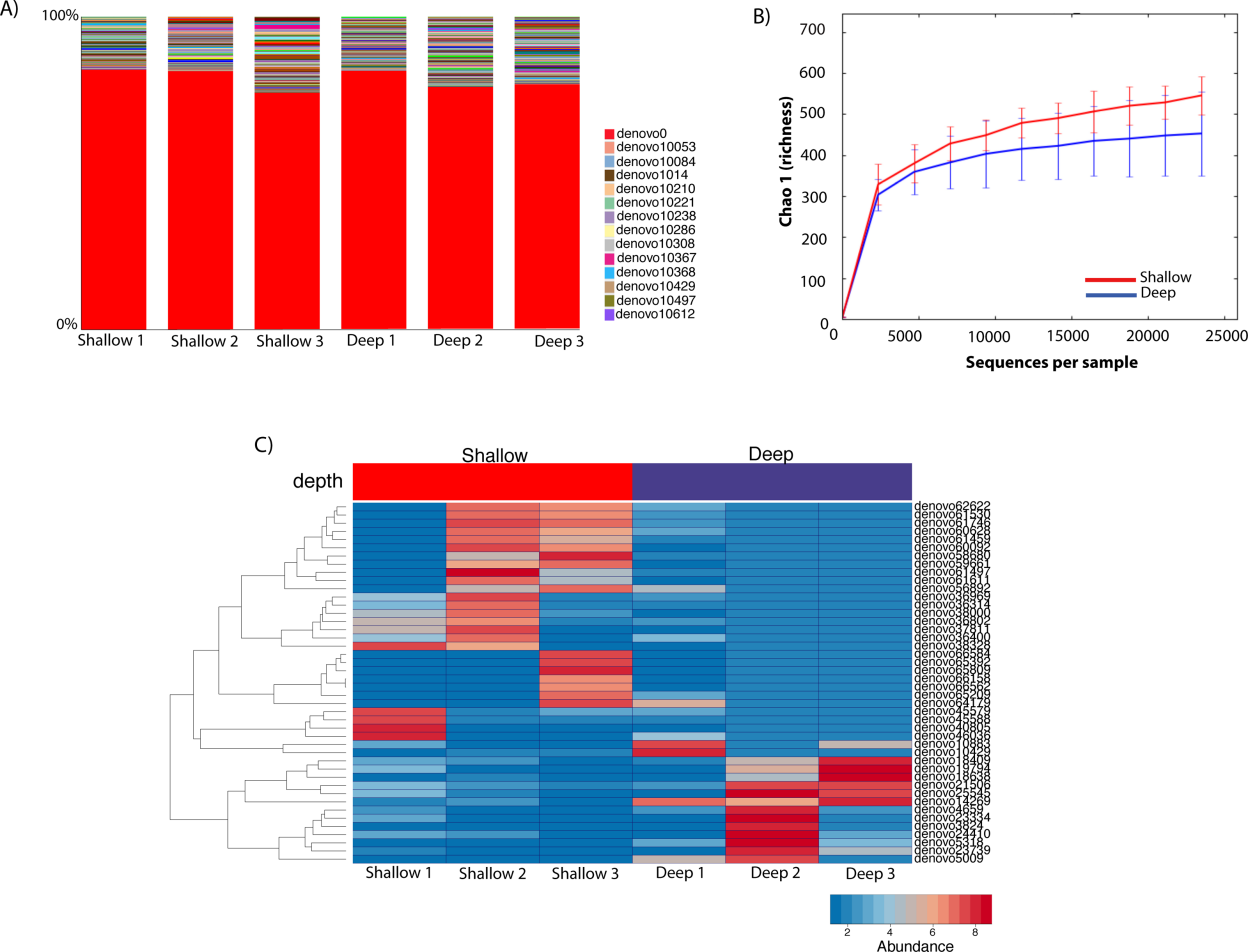
Taxa summary of the OTUs classified as Rickettsiales showing a dominant OTU (denovo_0) with a relative abundance of ∼80% and other hundreds of rare OTUs (A). Chao1 richness index of Rickettsiales populations between shallow and deep samples. Error bars represent standard deviation (*n* = 3 biological replicates/depth) (B). Heatmap of the 41 significantly different Ricketsiales OTUs (*p* < 0.00001) between shallow and deep water samples (C).

To gauge the diversity of the Rickettsiales-like OTUs, we performed a community profile analysis comparing Rickettsiales-like OTUs within shallow and deep samples using a rarefaction of 23,472 sequences (Fig. 4A). Out of the 725 Rickettsiales-like OTUs, a single OTU was most dominant across all samples (Fig. 4A, denovo_0). Furthermore, Rickettsiales richness was not significantly different between shallow and deep samples (*t*-test = 1.16, *p* = 0.226, Fig. 4B). As shown in Fig. 4B, the curve becomes asymptotic as the OTU number saturates, and each sample depth adds an increasingly smaller number of new OTUs, indicating adequate coverage for the environment. We did find that many Rickettsiales OTUs differ significantly in abundance between shallow and deep samples. For plotting purposes we selected those 44 OTUs whose *p* < 0.00001 (Fig. 4C).

### Differences in the microbiome of *A. cervicornis* corals inhabiting different depths

**Figure 5:**
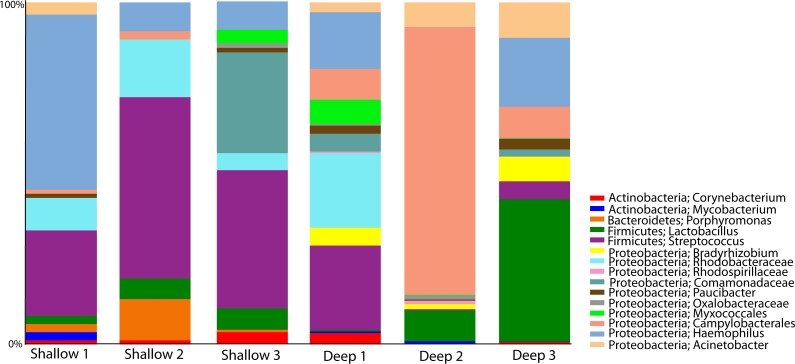
Bacterial OTUs shared amongst three to six coral samples, highlighting core taxa at both shallow and deep corals.

Once the microbial taxonomic profiles of *A. cervicornis* naturally inhabiting different depths was established, we next focused our analysis on those OTUs present in all three samples of one depth and absent in all the samples of the other depth (core depth analyses). This analysis revealed that the taxa shared by all shallow samples were mainly *Streptococcus, Haemophillus, Paucibacter* and *Porphyromonas* and in the deep samples the shared taxa were dominated by Campylobacterales, *Bradyrhizobium,* and *Lactobacillus*. Overall, 15 OTUs were plotted representing the core taxa, of these only four OTUs were shared between all six coral samples with all other being shared between more than three samples (Fig. 5).

**Figure 6:**
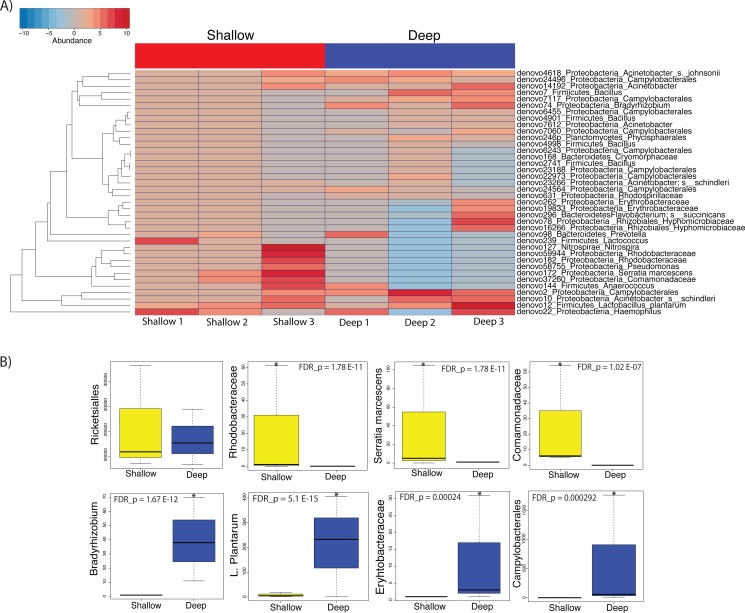
Heatmap showing the 38 significantly different taxa between shallow and deep samples (A). Boxplots of taxa found to be differentially abundant between depths (B). Such depth-driven differences were statistically significant for those marked by asterisks (false discovery rate-corrected *p* < 0.05), except for the Rickettsiales plot, appearing as example and validation of a taxa that did not change significantly according to depth.

We then proceeded to determine which taxa changed significantly (selected OTUs with *p* ≤ 0.05) between shallow and deep samples by employing a log-likelihood ratio test. Upon performing this analysis, we found that 38 OTUs varied significantly between shallow and deep samples (Figs. 6A and 6B). Among the taxa that varied significantly between depths we found unclassified Rhodobacteraceae, *Lactococcus*, Comamonadaceae and *Serratia* to be more abundant in the shallow samples as compared to deep samples (Figs. 6A and 6B top panel). Conversely, we found a significantly higher abundance of Campylobacterales and *Bradyrhizobium, L. plantarum* and Erythrobacteraceae in the deeper coral samples (Figs. 6A and 6B). Taken together, our data suggest that only low dominance taxa changed significantly in abundance across depths.

## Discussion

In this report, we have characterized the microbial communities associated with individuals of the coral *A. cervicornis* naturally inhabiting areas with different environmental conditions. Individuals inhabiting different depths (and that are under different light regimes) displayed homogeneity in the dominant microbiota. These results are consistent with previous findings that characterize individual coral specimens from the same species that even when collected from locations that are kilometers apart share most of the same associated microbial species (Littman et al., 2009). In previous studies, acroporid corals in particular, have displayed a dominance of Alpha-Proteobacterial Rickettsiales OTUs spread throughout healthy and diseased populations (Casas et al., 2004). The widespread and dominant Rickettsiales OTUs were confirmed in all the samples in this study with an unprecedented dominance of 95%, mainly from a single OTU (80% dominant).

On the other hand, we did observe statistically significant depth-related differences in the microbiota of *A. cervicornis* among taxa with less than 5% relative abundance. Amongst taxa that were significantly different between depths, *Serratia* was shown to be significantly more associated to shallow water samples. *Serratia* is known to be a human pathogen that has also been shown to contribute to mortality in the common Caribbean elkhorn coral *Acropora palmata* (Sutherland et al., 2011).

Coral samples from deeper waters displayed an abundance of *Lactobacillus plantarum*, which has been isolated from the marine environment before and are known to produce antimicrobial peptides that inhibit the growth of pathogens (Karthikeyan & Santosh, 2009). Additionally, deep samples displayed a relatively high abundance of *Bradhyrrizobium* and other bradhyrrizobiaceae OTUs, some of the most commonly occurring rhizobia that form symbioses in the nodules of legume plants (Lema, Willis & Bourne, 2012). These associations would appear to be responsible for increased nitrogen fixation at deeper depths. Likewise, the significant abundance of Campylobacterales, well-known for their metabolic influence in nitrogen cycling, may also contribute to increased nitrogen fixation in corals inhabiting deeper waters (Kern & Simon, 2009).

Rare species have been increasingly recognized as drivers of key functions in aquatic and terrestrial ecosystems despite their low abundance, as recently reported (Lynch & Neufeld, 2015; Jousset et al., 2017). Low-abundance taxa found in coastal systems, such as mangrove environments, were documented to be associated with key biogeochemical functions, such as CO2 flux and oxidation–reduction potential; this suggests that the metabolic activity of low-abundance microbes could serve as an early warning sign for environmental change (Chambers et al., 2016). Additionally another recent study showed the importance of low-abundance bacteria in peat soil microcosms being drivers of sulfate reduction-dependent degradation of fermentation products (Hausmann et al., 2016). In marine systems, rare taxa have been found to be more transcriptionally active (increasing their ribosome content) despite low abundance (Hausmann et al., 2016) which could well be similar in the coral holobiont. Nonetheless, experiments including single cell genomic and transcriptomic sequencing on specific coral-associated taxa are needed, especially those focused on furthering our knowledge of the microbiome associated with resilient *A. cervicornis* populations. This, coupled with physiology studies on the coral holobiont, will allow for a better understanding on the conserved genomic, metabolic and structural features that may be predictive of a physiological potential to resist climate change-associated environmental fluctuations.

The taxonomic diversity of microbes in association with different coral species has revealed a plethora of microbial functions important to the holobiont (Mayfield et al., 2014; Cardini et al., 2015). Although the number of samples in our study is too small to allow finding depth-related biomarkers, we have identified rare taxa significantly enriched in each depth, including nitrogen fixers in the deep corals, opening new avenues for research aiming at understanding how microbial communities and their metabolism relate to coral colony resilience.

Efforts to recover endangered coral species are largely based on finding resilient individuals that can be used for transplant restoration procedures (Van Katwijk et al., 2015). Resilience may be at least partly conferred by the microbiota of those individuals, and should be taken into account in these efforts. Characterizing the prokaryote microbiota of individuals inhibiting different water depths, including the identification of taxa in corals challenged with different light and temperature regimes, may help identify microorganisms that contribute to fitness for growth and reproduction, thus offering insights that may prove advantageous in coral restoration procedures.

## Conclusions

Although numerous sequence-based surveys of coral-associated microbiota have come to define common associations of the coral microbiota over time or geographical locations, this is likely the first report of the microbiota inhabiting *A. cervicornis* in Puerto Rico. Although we found only slight differences in the coral microbiota between depths, these corresponded to rare taxa that can have important metabolic activities in the coral holobiont. As sequencing prices continue to drop significantly and sequencing depth increases, the taxonomic characterization of the overlooked and rare taxa of the coral microbiota may become a useful and cheap approach to understand the microbial contributions to coral homeostasis and shed light on how coral species will cope with ongoing climate change.

##  Supplemental Information

10.7717/peerj.3717/supp-1 Table S1 OTU table with the 803 OTUs

## Additional Information and Declarations

### Field Study Permissions

The following information was supplied relating to field study approvals (i.e., approving body and any reference numbers): The Department of Natural and Environmental Resources of Puerto Rico approved this experiment.

### DNA Deposition

The following information was supplied regarding the deposition of DNA sequences: The resulting demultiplexed raw sequences per sample, as well as the 803 16S rRNA gene sequence representatives of the operational taxonomic units (OTUs) per sample were deposited in the NCBI BioProject ID PRJNA379103 with SRA accession SRP102061.

### Data Availability

The following information was supplied regarding data availability: NCBI SRA SRP102061: https://www.ncbi.nlm.nih.gov/sra/?term=SRP102061. A detailed repository and tutorial with all the necessary intermediary files and scripts to generate the plots and perform the analyses used in this publication is available on GitHub: https://github.com/meglab2017/The-microbial-biosphere-of-the-coral-Acropora-cervicornis-in-Northeastern-Puerto-Rico/projects.

## References

[ref-1] Ainsworth TD, Krause L, Bridge T, Torda G, Raina J, Zakrzewski M, Gates R, Padilla-Gamiño C, Spalding H, Smith C, Woolsey E, Bourne D, Bongaerts P, Hoegh-Guldberg O, Leggat W (2015). The coral core microbiome identifies rare bacterial taxa as ubiquitous endosymbionts. ISME Journal.

[ref-2] Aronson R, Precht WF (2001). White-band disease and the changing face of Caribbean coral reefs. Hydrobiology.

[ref-3] Bayer T, Neave M, Alsheikh-Hussain A, Aranda M, Yum L, Mincer T (2013). The microbiome of the Red Sea coral Stylophora pistillata is dominated by tissue-associated Endozoicomonas bacteria. Applied and Environmental Microbiology.

[ref-4] Bridge TC, Hoey AS, Campbell SJ, Muttaqin E, Rudi E, Fadli N, Baird AH (2014). Depth-dependent mortality of reef corals following a severe bleaching event: implications for thermal refuges and population recovery (version 3; referees: 2 approved, 1 approved with reservations). F1000Research.

[ref-5] Caporaso JG, Lauber CL, Walters WA, Berg-Lyons D, Huntley J, Fierer N, Owens SM, Betley J, Fraser L, Bauer M, Gormley N, Gilbert JA, Smith G, Knight R (2012). Ultra-high-throughput microbial community analysis on the Illumina HiSeq and MiSeq platforms. ISME Journal.

[ref-6] Cardini U, Bednarz VN, Naumann MS, Van Hoytema N, Rix L, Foster RA, Al-Rshaidat MM, Wild C (2015). Functional significance of dinitrogen fixation in sustaining coral productivity under oligotrophic conditions. Proceedings of the Royal Society B.

[ref-7] Casas V, Kline DI, Wegley L, Yu Y, Breitbart M, Rohwer F (2004). Widespread association of a Rickettsiales-like bacterium with reef-building corals. Environmental Microbiology.

[ref-8] Chambers LG, Guevara R, Boyer JN, Troxler TG, Davis SE (2016). Effects of salinity and inundation on microbial community structure and function in a mangrove peat soil. Wetlands.

[ref-9] Chao A (1987). Estimating the population size for capture-recapture data with unequal catchability. Biometrics.

[ref-10] Edgar R (2010). Search and clustering orders of magnitude faster than BLAST. Bioinformatics.

[ref-11] Faith DP (1992). Conservation evaluation and phylogenetic diversity. Biological Conservation.

[ref-12] Frias-Lopez J, Shi Y, Tyson GW, Coleman ML, Schuster SC, Chisholm SW, Delong EF (2008). Microbial community gene expression in ocean surface waters. Proceedings of the National Academy of Sciences of the United States of America.

[ref-13] Hausmann B, Knorr KH, Schreck K, Tringe SG, Glavina Del Rio T, Loy A, Pester M (2016). Consortia of low-abundance bacteria drive sulfate reduction-dependent degradation of fermentation products in peat soil microcosms. ISME Journal.

[ref-14] Hernandez-Delgado EA, Toledo-Hernández C, Claudio GH, Lassus J, Lucking MA, Fonseca J, Hall K, Rafols J, Horta H, Sabat AM (2006). Spatial and taxonomic patterns of coral bleaching and mortality in Puerto Rico during year 2005.

[ref-15] Holbrook SJ, Schmitt RJ, Messmer V, Brooks AJ, Srinivasan M, Munday PL, Jones GP (2015). Reef fishes in biodiversity hotspots are at greatest risk from loss of coral species. PLOS ONE.

[ref-16] Jousset A, Bienhold C, Chatzinotas A, Gallien L, Gobet A, Kurm V, Küsel K, Rillig M, Rivett D, Salles J, Van der Heijden M, Youssef N, Zhang X, Wei Z, Hol WH (2017). Where less may be more: how the rare biosphere pulls ecosystems string. ISME Journal.

[ref-17] Karthikeyan V, Santosh SW (2009). Isolation and partial characterization of bacteriocin produced from Lactobacillus plantarum. African Journal of Microbiology Research.

[ref-18] Kern M, Simon J (2009). Electron transport chains and bioenergetics of respiratory nitrogen metabolism in Wolinella succinogenes and other Epsilonproteobacteria. Biochimica et Biophysica Acta.

[ref-19] Kuczynski J, Stombaugh J, Walters WA, Gonzalez A, Caporaso JG, Knight R (2012). Using QIIME to analyze 16S rRNA gene sequences from microbial communities. Current Protocols in Microbiology.

[ref-20] Lee OO, Yang J, Bougouffa S, Wang Y, Batang Z, Tian R (2012). Spatial and species variations in bacterial communities associated with corals from the red sea as revealed by pyrosequencing. Applied and Environmental Microbiology.

[ref-21] Lema KA, Willis BL, Bourne DG (2012). Corals form characteristic associations with symbiotic nitrogen-fixing bacteria. Applied and Environmental Microbiology.

[ref-22] Levitus S, Antonov JI, Boyer TP, Locarnini RA, Garcia HE, Mishonov AV (2009). Global ocean heat content 1955–2008 in light of recently revealed instrumentation problems. Geophysical Research Letters.

[ref-23] Littman RA, Willis BL, Pfeffer C, Bourne DG (2009). Diversities of coral-associated bacteria differ with location, but not species, for three acroporid corals on the Great Barrier Reef. FEMS Microbiology Ecology.

[ref-24] Lozupone C, Hamady M, Knight R (2006). UniFrac—an online tool for comparing microbial community diversity in a phylogenetic context. BMC Bioinformatics.

[ref-25] Lynch MD, Neufeld JD (2015). Ecology and exploration of the rare biosphere. Nature Reviews in Microbiology.

[ref-26] Mayfield AB, Wang YB, Chen CS, Lin CY, Chen SH (2014). Compartment-specific transcriptomics in a reef-building coral exposed to elevated temperatures. Molecular Ecology.

[ref-27] McDonald D, Price MN, Goodrich J, Nawrocki EP, Desantis TZ, Probst A, Andersen GL, Knight R, Hugenholtz P (2011). An improved Greengenes taxonomy with explicit ranks for ecological and evolutionary analyses of bacteria and archaea. ISME Journal.

[ref-28] Mercado-Molina A, Ruiz-Diaz CP, Pérez ME, Rodriguez-Barreras R, Sabat AM (2015). Spatial–temporal population dynamics of the staghorn coral *Acropora cervicornis*: a threatened reef building species. Coral Reefs.

[ref-29] Morrow KM, Moss AG, Chadwick NE, Liles MR (2012). Bacterial associates of two Caribbean coral species reveal species-specific distribution and geographic variability. Applied and Environmental Microbiology.

[ref-30] Oksanen J, Kindt R, Legendre P, O’Hara B, Simpson GL, Stevens MHH, Wagner H (2008). https://CRAN.R-project.org/package=vegan.

[ref-31] Rohwer F, Seguritan V, Azam F, Knowlton N (2002). Diversity and distribution of coral-associated bacteria. Marine Ecology Progress Series.

[ref-32] Rosenberg E, Koren O, Reshef L, Efrony R, Zilber-Rosenberg I (2007). The role of microorganisms in coral health, disease and evolution. Nature Reviews Microbiology.

[ref-33] Ruiz-Diaz CP, Toledo-Hernandez C, Mercado-Molina AE, Perez ME, Sabat AM (2016). The role of coral colony health state in the recovery of lesions. PeerJ.

[ref-34] Santos HF, Carmo FL, Leite DC, Jesus HE, De Maalouf PC, Almeida C, Soriano AU, Altomari D, Suhett L, Volaro V, Valoni E, Francisco M, Vieira J, Rocha R, Sardinha BL, Mendes LB, Joao RR, Lacava B, Jesus RF, Sebastian GV, Pessoa A, Van Elsas JD, Rezende RP, Pires DO, Duarte G, Castro CB, Rosado AS, Peixoto RS (2012). Comparison of different protocols for the extraction of microbial DNA from reef corals. Brazillian Journal of Microbiology.

[ref-35] Sogin ML, Morrison HG, Huber JA, Mark Welch D, Huse SM, Neal PR, Arrieta JM, Herndl GJ (2006). Microbial diversity in the deep sea and the underexplored “rare biosphere”. Proceedings of the National Academy of Sciences of the United States of America.

[ref-36] Sun W, Anbuchezhian R, Li Z (2016). Association of coral-microbes, and the ecological roles of microbial symbionts in corals. The cnidaria, past, present and future.

[ref-37] Sunagawa S, DeSantis TZ, Piceno YM, Brodie EL, DeSalvo MK, Voolstra CR, Weil E, Andersen GL, Medina M (2009). Bacterial diversity and White Plague Disease-associated community changes in the Caribbean coral Montastraea faveolata. ISME Journal.

[ref-38] Sutherland KP, Shaban S, Joyner JL, Porter JW, Lipp EK (2011). Human pathogen shown to cause disease in the threatened eklhorn coral *Acropora* palmata. PLOS ONE.

[ref-39] Thompson JR, Rivera HE, Closek CJ, Medina M (2014). Microbes in the coral holobiont: partners through evolution, development, and ecological interactions. Frontiers in Cellular and Infection Microbiology.

[ref-40] Van Katwijk MM, Thorhaug A, Marb AN, Orth RJ, Duarte CM, Kendrick GA, Althuizen IJ, Balestri E, Bernard G, Cambridge ML, Cunha A, Durance C, Giesen W, Han Q, Hosokawa S, Kiswara W, Komatsu T, Lardicci C, Lee K-S, Meinesz A, Nakaoka M, O’Brien KR, Paling EI, Pickerell C, Ransijn A, Verduin J (2015). Global analysis of seagrass restoration: the importance of large-scale planting. Journal of Applied Ecology.

[ref-41] Vazquez-Baeza Y, Pirrung M, Gonzalez A, Knight R (2013). EMPeror: a tool for visualizing high-throughput microbial community data. GigaScience.

[ref-42] Vega Thurber R, Willner-Hall D, Rodriguez-Mueller B, Desnues C, Edwards RA, Angly F, Dinsdale E, Kelly L, Rohwer F (2009). Metagenomic analysis of stressed coral holobionts. Environmental Microbiology.

[ref-43] Wickham H (2007). Reshaping data with the reshape Package. Journal of Statistical Software.

[ref-44] Zhao S, Guo Y, Sheng Q, Shyr Y (2014). heatmap3: an improved heatmap package. BMC Bioinformatics.

